# Fangs for the Memories? A Survey of Pain in Snakebite Patients Does Not Support a Strong Role for Defense in the Evolution of Snake Venom Composition

**DOI:** 10.3390/toxins12030201

**Published:** 2020-03-22

**Authors:** Harry Ward-Smith, Kevin Arbuckle, Arno Naude, Wolfgang Wüster

**Affiliations:** 1 Molecular Ecology and Fisheries Genetics Laboratory, School of Natural Sciences, Bangor University, Bangor LL57 2UW, UK; harry_ws@hotmail.co.uk; 2 Department of Biosciences, College of Science, Swansea University, Swansea SA2 8PP, UK; kevin.arbuckle@swansea.ac.uk; 3 Snakebite Assist, Pretoria ZA-0001, South Africa; afnaude@gmail.com

**Keywords:** Defense, evolution, pain, selective pressure, snake, snakebite, survey, venom

## Abstract

Animals use venoms for multiple purposes, most prominently for prey acquisition and self-defense. In snakes, venom composition often evolves as a result of selection for optimization for local diet. However, whether selection for a defensive function has also played a role in driving the evolution of venom composition has remained largely unstudied. Here, we use an online survey of snakebite victims to test a key prediction of a defensive function, that envenoming should result in the rapid onset of severe pain. From the analysis of 584 snakebite reports, involving 192 species of venomous snake, we find that the vast majority of bites do not result in severe early pain. Phylogenetic comparative analysis shows that where early pain after a bite evolves, it is often lost rapidly. Our results, therefore, do not support the hypothesis that natural selection for antipredator defense played an important role in the origin of venom or front-fanged delivery systems in general, although there may be intriguing exceptions to this rule.

## 1. Introduction

*“Bee stings hurt. So do wasp stings, scorpion stings, the bites of centipedes, and the venom injections of many other animals, including snakes. To inflict pain is not necessarily to the advantage of an animal that uses its venom strictly for incapacitation of prey. In fact, it may be to its disadvantage because pain may induce increased struggling on the part of the prey. But venoms are also used defensively, and it is in that context that they may derive their effectiveness largely, if not exclusively, from their pain-inducing qualities. It is principally because venoms are painful that they can function in defense”.*

—Eisner and Camazine [1]

Venoms are widespread across the animal kingdom, and have evolved numerous times in a broad range of phyla [2], with further examples still being discovered regularly, such as venomous crustaceans [3] and frogs [4]. The biological functions of venomous secretions include primarily predation and anti-predator defense, as well as intraspecific competition, reproduction, and digestion [2,5]. While a primary function can be identified for most venom systems, many venomous animals use their venoms for multiple purposes. In particular, animals with primarily foraging venoms frequently employ these for anti-predator defense [2].

Among venomous animals, snakes have received the greatest amount of research attention, due to their medical significance [6], and because the large volumes of venom secreted by many species greatly facilitate toxicological research. Snake venoms are highly variable in composition at all taxonomic levels, from ontogenetic variation within individuals [7] to geographic variation within species [8] and differences between higher taxa. The mechanisms and selective drivers of this variation have attracted extensive research attention.

Snakes use their venoms for both foraging and self-defense, but the relative importance of these as drivers of venom evolution has remained poorly understood. The “life-dinner principle” [9] suggests that defense, where the snake is fighting for its life, should take precedence over foraging efficiency, where a suboptimal strategy would merely result in reduced energy intake. However, most of the literature on the selective drivers shaping venom composition has focussed on the role of diet. 

Studies in multiple taxa and using diverse approaches have accumulated a considerable body of evidence that many snake venoms have evolved under selection to optimize their prey-specific toxicity. Diet-related evolutionary effects were first discovered through correlations between venom composition and diet in *Calloselasma rhodostoma* [10]. Direct functional evidence in the shape of prey-specific lethality has been demonstrated on multiple occasions. For instance, the venoms of naturally arthropod-eating species of *Echis* and *Vipera* are more toxic to invertebrate prey than those of predominantly vertebrate-feeding congeneric species [11,12,13]. Prey-specific venom toxicity has also been detected in the venoms of different species of *Sistrurus* [14], and across multiple species of New World coral snakes (*Micrurus*) [15]. Among colubrid venoms, individual toxins with specific toxicity to avian and lizard prey have been documented in *Boiga* spp. [16,17,18], *Oxybelis fulgidus* [19] and *Spilotes sulphureus* [20]. Patterns of ontogenetic variation in venom composition in vipers have also been found to reflect ontogenetic diet changes [21]. Moreover, many prey species have evolved various levels of resistance to snake venoms [22,23,24], resulting in a toxic arms race that has led to prey-specific venom evolution in the snakes [25]. While the link between diet and venom composition may not be universal (e.g., Zancolli et al. [26]), the idea that venom composition is driven primarily by selection for prey subjugation has become the dominant paradigm in snake venom evolution. 

While the venom system of most non-front-fanged snakes is of limited effectiveness against predators [27], we know from the global impact of snakebite that front-fanged venomous snakes frequently use their venoms in self-defense, often to devastating effect. This is supported by the evolution of highly specific defensive adaptations, such as hooding, tail vibration, scale rubbing and the rattle [28]. The frequent evolution of venom resistance among snake predators [25,29], predator avoidance of front-fanged snakes [30,31], the evolution of innate avoidance of characteristic venomous snake colour patterns [32,33], and the evolution of Batesian mimicry of front-fanged snakes [34,35] all indicate that venom can be an effective defense against at least some predators. However, whereas adaptation of venom composition to natural prey has become a well-documented phenomenon, we remain largely ignorant whether natural selection for defensive purposes may also have played a role in driving venom composition [36]. Harry W. Greene recognized this deficit in 2013 [28] predicting that “we’ll soon be asking if toxins had more to do with defense than heretofore realized”. 

To test for selection for a defensive function, it is essential to first consider the requirements for a defensive venom: for a venom to be effective in that role, it must repel a predatory attack sufficiently rapidly for its producer to escape serious injury or death. This is most readily achieved through the rapid infliction of pain beyond that expected from the physical trauma of the bite alone [1,37]. In human patients, these characteristics are evident from clinical cases involving many primarily defensive animal venoms. For instance, virtually all venomous fish use their potent venoms solely for defense, invariably causing intense pain immediately upon envenomation [38,39,40,41,42]. Similarly, the entirely defensive venoms of non-predatory hymenopterans such as honeybees are equally notable for the immediate pain following the sting. Other invertebrates that use their venom for both predation and defense nevertheless include specifically pain-inducing toxins in their venom. This includes many scorpions [43] and centipedes of the genus *Scolopendra* that produce symptoms which, although rarely fatal to humans, are characterized by intense pain immediately upon envenomation, caused by a specific pain-causing toxin [44]. These offer examples of venom which are highly effective both in predatory and defensive contexts. 

Whereas rapid-onset pain is ubiquitous and well documented in the examples of clearly defensive venoms, we lack systematic information on pain after snakebite. It is widely acknowledged that snakebites often entail significant or extreme pain [45,46,47]. However, the timeframe of its development is rarely stated. From anecdotal reports, we know that bites by many species result in great variation in the level and time course of pain experienced, with some bites resulting in immediate intense pain while others cause none [48]. Moreover, pain often appears to be a delayed symptom secondary to other venom effects, such as severe swelling or local tissue destruction [48]. Indeed, some snakes are notorious for the lack of early pain caused by their bites: for instance, in *Bungarus* envenomations, which often occur while the victim is asleep, initial pain is often never felt [49].

Limited evidence exists of specific pain-inducing toxins in certain species. Bohlen et al. [50] discovered the first snake venom toxin to specifically cause pain in the venom of *Micrurus tener*. MitTx was found to have no other function than to activate acid-sensing ion channels (ASICs), producing pain. MitTx has subsequently also been found in the venom of *M. mosquitensis* and *M. nigrocinctus* [51], but interestingly, the closely related *M. fulvius* lacks MitTx [52,53], indicating that this pain-inducing toxin is phylogenetically labile within *Micrurus*. More recently the Lys49 myotoxin BomoTx, found in the venom of *Bothrops moojeni*, was discovered to induce intense pain [54] through the promotion of ATP release, which consequently activates the P2X2 and/or P2X3 purinergic receptors. However, the relationship between the presence or absence of these toxins and the actual pain experienced by bitten adversaries has not been explored.

The very limited data currently available on the ability of different snake venoms to cause early pain post-bite restricts our ability to infer the role of antipredator defense in driving the evolution of snake venom composition. The assessment of pain from envenomation is potentially complicated by taxonomic differences in nociceptor function and pain perception. However, the structure of nociceptors appears to be highly conserved across both vertebrates and invertebrates, as does the central processing of nociception, which gives rise to the perception of pain [55]. There are exceptions to these rules, such as the lack of sensitivity to capsaicin in birds or to acidity in naked mole rats [55], and specific resistance in some specialized predators of venomous organisms, such as the specific blocking of scorpion venom-induced algesia documented in scorpion-feeding grasshopper mice (*Onychomys* sp.) [43]. However, it seems highly likely that most predators are likely to show similar patterns of nociceptor activation in response to venomous challenges, especially in terms of their time-course. This also suggests that the pain experience of a human snakebite patient is likely to be representative of that of other generalized predators.

Since the testing of nociceptor activation in the laboratory is time-consuming and may be difficult to relate to the perceived level of pain in vivo [44], we sought instead to assess the defensive potential of different snake venoms by using human snakebite victims as a model system that allows data on pain perception to be recalled and directly communicated. An increasing number of humans interact regularly and intentionally with venomous snakes in a professional capacity or as part of leisure activities, and as a result, numerous bites by a wide variety of snake species occur every year [56,57,58]. These well-informed bite victims represent a potentially valuable source of information on snakebite symptoms, as they are capable of providing positive identification of the snake species, and, due to their awareness of the risks of their activities, they are likely to be on average less susceptible to fear-induced memory distortions than unprepared victims of entirely unexpected ‘accidental’ bites. The large body of collective experience of snakebites among reptile workers thus represents an unparalleled source of information on the development of pain after snakebite.

Here, we exploit this collective reservoir of knowledge through the use of a questionnaire that seeks to establish the severity and, more importantly, the time course of pain development in patients envenomed by a diversity of snake species spanning the phylogenetic breadth of venomous caenophidians. We postulate that any venom at least partly shaped by selection for antipredator defense should cause pain of rapid onset to deter a predator in the critical early stages of any encounter, potentially giving the snake a chance to escape before being seriously injured or killed. While the presence of early pain after a bite does not necessarily indicate adaptation to a primarily defensive function, absence of early pain would preclude such a role. We also predict that any at least partly defensive venom should generate a trajectory of pain that would be consistent between patients: although the perceived intensity of pain from a bee sting may vary between individuals, they invariably cause early pain, and the same would be expected of other defensive venoms. The aim of this survey is thus not to compare absolute pain levels across snake species, but instead to begin to understand the pain trajectory as an ecologically informative attribute of snakebite in the context of defense. 

## 2. Results

**Figure 1:**
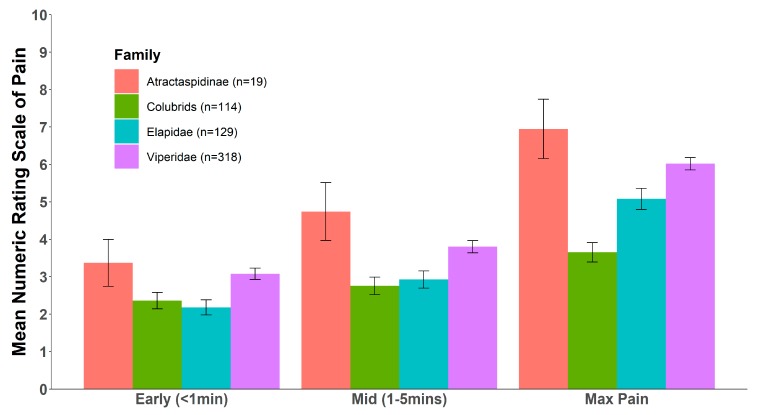
Mean pain Numeric Rating Scale (NRS) (on a 0–10 scale) across four major clades of venomous snakes within the first minute after the bite, within 1–5 min after the bite, and the maximum pain experienced at any time.

**Figure 2:**
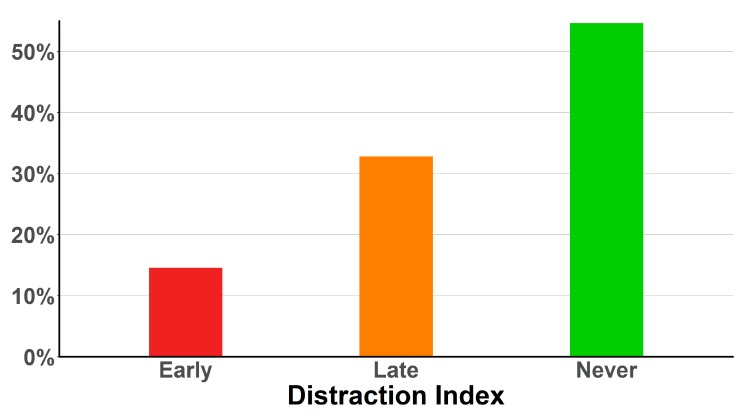
Percentage of bites where pain became too intense/distracting to continue with intended/normal activities across three time periods; early (within 5 min), late (after over 5 min), and never.

**Figure 3:**
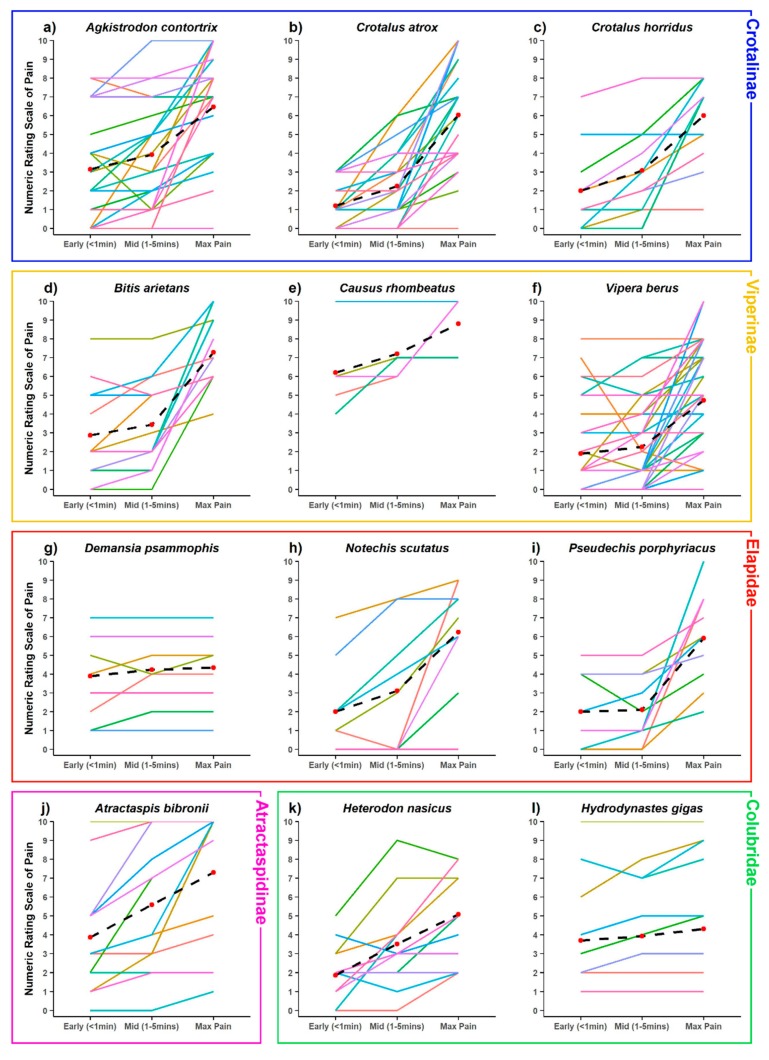
Variation shown of NRS at early, mid and max levels of pain of some of the best-sampled species, and species with unusual patterns. Data plotted of 11 point NRS, where 0 = no pain felt at all and 10 = the maximum level of pain imaginable. Crotalinae: (**a**) *Agkistrodon contortrix* [*n* = 28], (**b**) *Crotalus atrox* [*n* = 24], (**c**) *Crotalus horridus* [*n* = 13]; Viperinae: (**d**) *Bitis arietans* [*n* = 14], (**e**) *Causus rhombeatus* [*n* = 5], (**f**) *Vipera berus* [*n* = 40]; Elapidae: (**g**) *Demansia psammophis* [*n* = 9], (**h**) *Notechis scutatus* [*n* = 9], (**i**) *Pseudechis porphyriacus* [*n*=9]; Atractaspidinae: (**j**) *Atractaspis bibronii* [*n* = 14]; Colubridae: (**k**) *Heterodon nasicus* [*n* = 14], (**l**) *Hydrodynastes gigas* [*n* = 13]. Black dashed lines plot the mean trajectory and value at each time point. Note the relatively flat lines in *Causus rhombeatus*, *Demansia psammophis* and *Hydrodynastes gigas*, indicating a relatively early onset of pain.

The distribution of sex to age of the 584 individual bite reports received in this study are shown in Table 1. In all snake families, mean pain levels within one and five minutes after the bite were considerably lower than the maximum pain level reported in the later phases of envenoming (Figure 1). The pain became too distracting for normal activities within the ecologically crucial first 5 min in only 14.55% of bite victims, and later than 5 min in another 30.82% (Figure 2). Remarkably, 54.62% reported never experiencing pain great enough to make normal activities impossible. Moreover, the pain experienced by different individuals bitten by the same species varied immensely, not only in its absolute level but also in its trajectory. Figure 3 shows the mean and individual pain trajectories for 12 representative and well-sampled species from all snake families. While absolute pain levels are likely to vary subjectively, the trajectory of pain development also varied extensively within many species (e.g., *Agkistrodon contortrix*, *Vipera berus*, *Atractaspis bibronii*), but was much more consistent in others (e.g., *Crotalus atrox*, *Bitis arietans*) (Figure 3).

**Figure 4:**
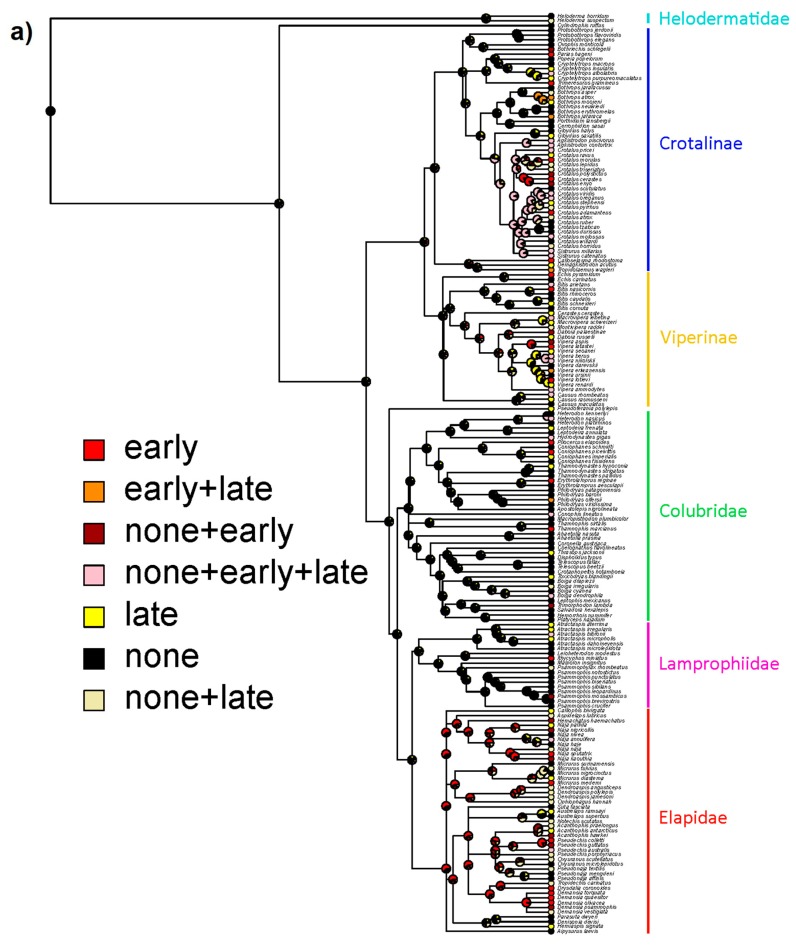

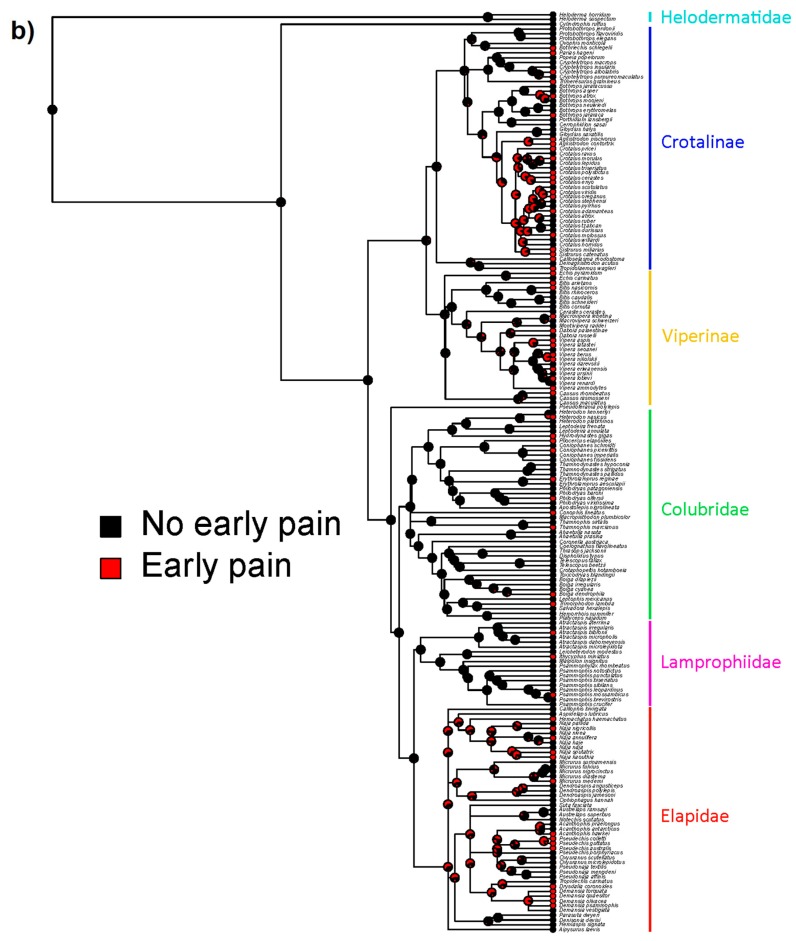
Ancestral state estimates for pain trajectories showing either full coding of each individual combination of states (**a**) or simplified into either inducing early pain (with or without variation) or not (**b**). Venoms that cause early pain occur frequently across the current diversity of venomous reptiles but with two major exceptions (Elapidae and New World pitvipers) are mostly independent origins.

Consistent with these results, and despite the phylogenetically widespread nature of envenomations causing early pain, our ancestral state estimates suggest that the majority of the history of venomous reptiles has been characterized by venoms causing little pain, particularly no early pain (Figure 4). Nevertheless, there are two prominent exceptions to this pattern: Elapidae and the New World radiation of pit vipers. Interestingly we estimate that these two deeper origins of early-pain-inducing venoms arose in different ways. In the ancestor of elapid snakes, the venom most likely caused early pain with little intraspecific variation, whereas in New World pit vipers intraspecific variation consisting of all three possible states (no, early, and late pain) is the estimated ancestral state (Figure 4). The estimated transition rates between states also suggest little evidence for a pervasive influence of a defensive function over the evolutionary history of venomous reptiles in general (Table 2). Specifically, states which include early pain (with or without intraspecific variation) tend to have higher transition rates which involve loss of early pain, suggesting it is not maintained by strong selection. Note that transition rates are not clade-specific but apply across the whole tree, so they do not preclude an effect of antipredator defense in particular clades (such as elapids as highlighted above), but suggest limited influence of defense in general.

**Figure 5:**
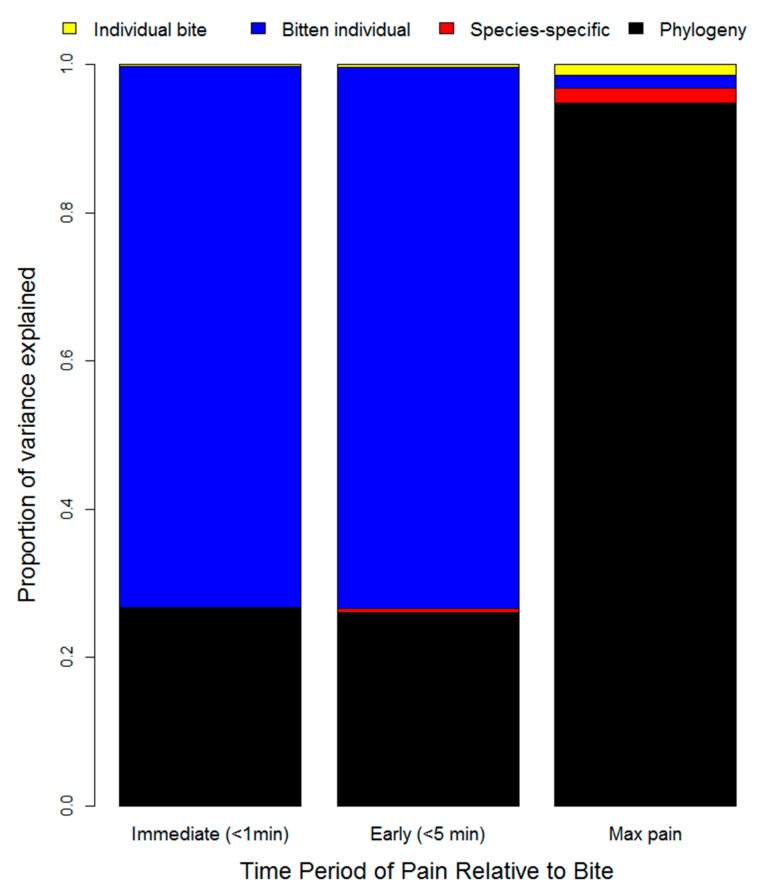
Variance in the magnitude of immediate, early, and maximum pain experienced by snakebite victims as explained by variation between individual bites, individual victims, snake species, and snake phylogeny. Note that individual bites are only a minor source of variation after accounting for the snake and the bitten individual, and there is a stark difference between variance components of the level of pain within the first 5 min (which mostly varies based on the bitten individual) and the maximum level of pain experienced over the course of the envenomation (which is mostly related to the phylogenetic history of the snake species involved).

The results from our variance partitioning analysis (using phylogenetic mixed models) suggest that most of the variation in levels of pain depends on the bitten individual (for immediate and early pain) and the phylogenetic history of the snake species which inflicted the bite (for the maximum pain resulting from the bite) (Figure 5). Phylogeny had a much stronger influence on the severity of pain than species-specific effects, which suggests that particular clades have characteristic venom compositions that influence the level of pain experienced from a bite. Nevertheless, despite explaining ~95% of the variance in maximum pain throughout the bite, any influence related to the snake responsible for the bite is relatively minor (~25%) for early pain-induction compared to victim characteristics (~75%). Because early pain is likely to be a key component of a defense-driven venom, our results suggest that, although there may be important differences between different clades of snakes, the overall evidence of selection for defense is limited. Note that we did find the predicted consistency across individual bites, which explains almost none of the variation (~0.3% for early pain and ~1.5% for maximum pain; Figure 5), but if we assume that humans are sufficiently analogous to other predators then the effect of the individual bitten suggests that early pain is likely to be particularly severe only in some bitten individuals.

## 3. Discussion

In summary, our results provide little evidence of pervasive selection for a defensive function in the evolution of snake venoms. The overall pattern from envenomed bites suggests that the majority of bites cause relatively little early pain, compared to the much higher levels of pain experienced later during the course of the envenoming. Strikingly, in the vast majority of bites sustained by our respondents, pain only became too distracting for other activities much later than during the first few minutes, and, even more surprisingly, in 54.62%, this never happened. This suggests that the venoms of these snakes would be ineffective in deterring a continued attack by a predator within an ecologically relevant timeframe.

Moreover, respondents bitten by some species had pain experiences that cannot be attributed solely to inter-individual differences in pain sensitivity, but that instead suggest intraspecific differences in venom activity. Even though we only considered bites with evidence of envenoming, some respondents bitten by species such as *Crotalus atrox*, *Vipera berus* and *Notechis scutatus* reported no pain whatsoever in the early or even later stages of envenoming, while others reported a strong later increase in pain, or even high early pain levels (Figure 3). Even accounting for individual differences in pain sensitivity, these extreme differences are difficult to reconcile with being due to identical venoms. Instead, they suggest intraspecific variation in venom composition with regard to algesic activity. This would be unexpected in a scenario of pervasive selection for a defensive function.

Similarly, the phylogenetic comparative analyses found little support for strong selection for a defensive function across the clade as a whole, and certainly not early in the caenophidian (or toxicoferan) radiation. Early pain as a consequence of venom appears to have evolved repeatedly, in particular, we find evidence for deeper origins at the base of the Elapidae and the New World pitvipers (with these deeper origins being far less likely to be explained by noise in the data). The evolution of consistent early pain in the Elapidae may be related to elapid venoms being typically more neurotoxic and so potentially targeting pain receptors directly (either as a directly selected or exapted effect), whereas in vipers the pain may be the result of SVMPs or similar toxins breaking down tissue and so is under weaker (if any) direct selection and is consequently more variable. This interpretation is consistent with our results from Figure 4, as the origin of early pain in elapids is estimated to be fairly consistently early pain, whereas in New World pitvipers it is estimated that bites could variably cause early, late, or no pain. If true, it suggests that elapid snakes are the best clade upon which to focus future efforts on understanding defense-driven evolution of pain. It also opens the intriguing possibility that spitting cobras (as the only snakes with unambiguously defensive adaptations of venom use) may have been exapted for defensive use of venom via early-pain inducing elapid ancestors.

Our estimated transition rates for pain trajectories find that the rate of loss of early pain was systematically higher than its rate of gain. This again suggests a lack of widespread selection pressure for a defensive function, as the early pain necessary for defense both evolves (relatively) infrequently and seems to have little selection pressure maintaining it when it does.

While the general pattern argues against a pervasive selection for defense, some taxa with divergent patterns such as relatively flat pain trajectories are worth noting, in particular *Causus rhombeatus*, *Hydrodynastes gigas* and especially *Demansia psammophis* (Figure 3). To identify outliers with potentially more defensive venoms, we explored the onset of incapacitating pain in the better-sampled species (N ≥ 5). Out of these 34 species, only four caused early incapacitating pain in more than 40% of all cases, andin more than 50% of those cases in which incapacitating pain occurred at all: *Causus rhombeatus*, *Agkistrodon piscivorus*, *Pseudechis australis* and the combined bites of all *Demansia* species. The individual variation in pain perceptions in the early stages of a bite may be relevant here as pain may only be experienced as incapacitating by a minor-moderate proportion of victims. In principle this could still lead to effective defense against some proportion of the predator community, but studies on variability in pain responses in natural predators are needed to further examine this possibility. In any case, even if effective against some predators, the inconsistency of the results adds to the weight of evidence against a strong role for defense in snake venom evolution in general. Moreover, we also stress again that early pain is necessary, but not sufficient, to infer selection for a defensive function, since it may also represent a mere side-effect of another venom activity.

**Figure 6:**
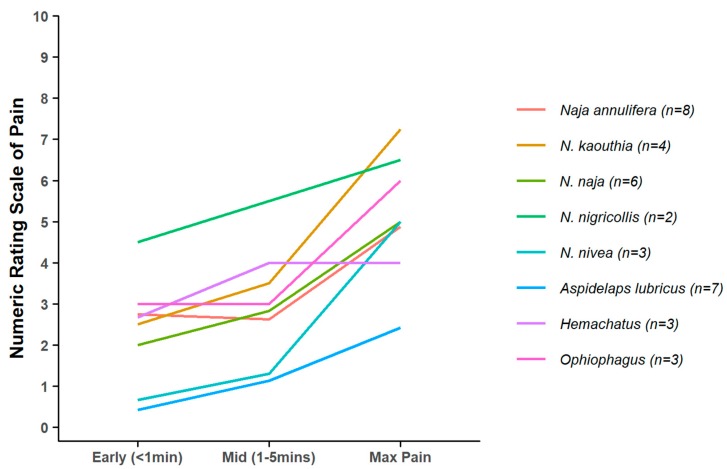
Mean pain trajectories for species of *Naja* and relatives. Note typical pain trajectory with relatively low early pain compared to the maximum later pain levels. Species with *n* = 1 were excluded.

In one of the few studies explicitly addressing the relationship between venom toxicological function and defensive adaptations, Panagides et al. [59] noted an association between the defensive adaptations of cobras (*Naja*) and relatives and venom cytotoxicity. They interpreted cytotoxicity as a defensive adaptation, on the assumption that it would be associated with greater pain. Our data do not support this inference, as the average pain trajectories of all *Naja* species in our dataset (but not *Hemachatus*) display the typical pattern of much lower pain in the first five minutes after the bite than later (Figure 6). Out of 26 *Naja* bites (all species), only four (15%), each by a different species, resulted in early incapacitating pain, and 19 (73%) never reached that pain level. While the sample sizes for the individual species are small, the emerging pattern does not support strong selection for a defensive function, contrary to the interpretation of Panagides et al. [59]. This may be because the assumption that cytotoxicity is a good proxy of early pain in cobras is incorrect. The clearest example of adaptations for defensive use of snake venoms is in venom spitting in cobras, which suggests that spitting cobras should cause more rapid early pain thannon-spitting species. Unfortunately, our sampling is insufficient to determine whether this is the case.

In contrast to the overall conclusions of this study, the evolution of specifically nociceptor-targeted toxins, such as BomoTX in *Bothrops moojeni* [54] and MitTX in *Micrurus tener* [50], strongly suggests a defensive function in those species. However, at least in coral snakes, the phylogenetically inconsistent distribution of this toxin argues against consistently strong selection for defense in this clade: MitTX is present in *M. tener*, *M. nigrocinctus* and *M. mosquitensis*, but absent in *M. fulvius*, the sister species of *M. tener*. A similar dimeric toxin is also present in the more distantly related *M. dumerili* and *M. frontalis* [60], and apparent homologues have been found in additional venoms [61], but again without a clear phylogenetic pattern.

The effect of these specifically algesic toxins on pain levels and trajectories in vivo remains largely unexplored. In a series of 39 bites by *M. fulvius*, which lacks MitTX, local pain appeared to be largely absent [62]. However, in another study [63], at last one patient bitten by the same species reported radiating pain. In comparison, 42.7% of 82 *M. tener* bites in Texas were followed by local pain (on an unknown timescale), but this was severe enough to require analgesia in only 15.9% [64]. A number of otherwise symptomatic patients in the latter series did not report pain, suggesting a lack of the kind of consistent pattern of early pain following fish or honeybee envenoming. Our sample of *Micrurus* bites is insufficient to add to this discussion, except to note that two bites by *M. nigrocinctus* resulted in little early pain.

Another factor arguing against pervasive selection for defense is the atrophy of the venom apparatus in snakes feeding on undefended prey. Among non-front-fanged snakes, the bird egg specialist *Dasypeltis* is phylogenetically nested in a clade of venomous opisthoglyphous genera such as *Boiga* and *Telescopus* [65], but its venom apparatus is atrophied [66]. Among front-fanged snakes, several elapid lineages that have specialized on the consumption of fish eggs (*Emydocephalus* spp. and *Aipysurus eydouxii*) display a greatly reduced venom apparatus and a series of deleterious mutations in their main toxin genes [67,68,69]. This suggests that, in the absence of a foraging function, there were no further selective pressures for the retention of a venom apparatus.

Inevitably, studies like the present one, that are based on the recollections of individuals that lived through a potentially traumatic experience, are likely to result in noisy data with multiple potential sources of error. These include faulty memory, subjective biases, individual differences in pain perception and tolerance, misidentification of snakes, and noise from a wide variety of unknown factors, such as site of bite, quantity and depth of venom injection, individual venom variation etc. Nevertheless, retrospective reports of pain intensity are commonly used and, given sufficient sample sizes, are often sufficiently reliable for epidemiological studies (e.g., Brauer et al. [70]).

Despite the inevitable statistical noise in survey data, they have allowed us to exploit the large body of collective experience accumulated by the herpetological community to assess the algesic properties of snakebites across a considerable breadth of snake diversity in a manner unachievable by other means. They have yielded a strongly supported and consistent pattern of limited early pain after snakebites, compared to higher maximum pain later, and a lack of early incapacitation from pain. This study thus adds to the evidence that venom in snakes has evolved for primarily foraging purposes and suggests that any effectiveness as a defensive adaptation is restricted to particular cases rather than a general (or early) driver of venom evolution.

This leads to the question of why, against the predictions of the life-dinner principle, selection for defense did not play a greater role in the evolution of snake venom. It may be that for the most part, biting is the final strategy in a snake’s defensive arsenal, because contact with the predator increases the risk of injury to the snake [71]. To reduce the necessity for this risk, snakes have evolved other defensive strategies that they employ before biting to deter and evade predation [72,73]. The evolution of behaviour to utilise alternative defensive strategies prior to biting may have reduced the selective pressures of defense upon the composition and toxicological effects of snake venoms.

Another reason may lie in the extremely lethal power of many front-fanged venomous snakes [74]. Numerous venomous organisms, such as insects and most fish, rely on painful rather than lethal venomous defenses, where individual predators are deterred by pain resulting from individual stings, and each sting needs to cause pain to generate that deterrence. Front-fanged venomous snakes have sufficient lethal potential to incapacitate or kill many predators. As a result, rather than relying on deterrence of individual predators through pain, deterrence may also develop through social learning in some predators witnessing the death, suffering or incapacitation of a conspecific or relative [34,75], or through natural selection for innate avoidance [32,33]. Neither of these mechanisms requires early pain or other specifically defensive adaptations of venom composition, but mathematical models have suggested instead that a quantitative increase in lethality may be selected for under some scenarios [76].

Despite the lack of pervasive selection for defense revealed here, the role of snake venom in antipredator defense, and the ecological and evolutionary factors that may influence such interactions, remain potentially rewarding subjects for further investigation. Currently, we lack even the most basic quantitative data on the use of venom in interactions between snakes and their predators, including any indication of how often snakes ever employ venom defensively, and how frequently this use of venom affects the outcome of these encounters. Although our current study suggests that defense has not been the primary driver of snake venom evolution in general, particularly early in the history of the clade, we also suggest that some exceptions may exist in certain groups. Moreover, groups that diverge from the majority of snakes in their use of venom, especially spitting cobras, may represent rewarding targets for more detailed investigations of when, why, and how antipredator defense might act as an important factor in snake venom evolution.

## 4. Materials and Methods

### 4.1. Questionnaire and Data Collection

To obtain data on the collective experience of snakebite pain from the herpetological community, we designed a questionnaire to chart the time course of pain development in envenomations. Respondents were asked to identify the species of snake they were bitten by and rate their level of pain experienced at three time periods: immediate (within 1 min post-bite), early (1 to 5 min post-bite) and later (maximum level of pain), utilizing an 11 point (0–10) Numeric Rating Scale (NRS) to give the most reliable account of pain experienced [77,78,79]. Respondents were then asked when the pain became ‘too distracting’ (distraction index), defined as how long after the bite the level of pain became too intense to continue with intended/normal activities: early (subgroups of: immediately, <1 min and 1 to 5 min), late (>5 min), or never.

Other questions related to the sex and age of the respondent at the time of the bite, the site of the envenomation on the body, and the sex, size and life stage of the snake responsible. No other medical symptoms were asked for, but many participants chose to include them, within the subsequent comment section. Where a respondent had received multiple envenomed bites, separate reports were collected for each event. Bite reports from both wild (51.2%) and captive (48.8%) snakes were included in the analyses. “Dry” bites without clinical symptoms of envenoming were excluded.

Professional herpetologists, herpetoculturists and herpetological fieldworkers were targeted to reduce the variance in pain perception in reports. To reach the largest audience of herpetologists possible, the questionnaire was created and distributed electronically using Google Forms (https://goo.gl/forms/A8FdnjVRqMnUnV162). The survey became publicly available in November 2016 and was extensively advertised via e-mail and shared on over 130 herpetological Facebook groups and via Twitter. Here, we analyze responses to the questionnaire received until December 2018. Bites were excluded from our analyses where the victim, at the time of the bite, was <10 years of age, or where a person submitted a report of snakebite sustained by a third party. After removal of reports based on the above criteria, and obviously erroneous or facetious entries, our final dataset used in further analyses contained reports of 584 individual bites, inflicted on 368 individual respondents by a total of 192 snake species.

Inevitably, self-reported, survey-based measures of pain will contain substantial statistical noise. However, as there is no obvious a priori reason to expect bias (systematic errors in a direction that are likely to mislead attempts to answer our specific questions), such noise is likely to be random. The effect of this should simply be to reduce the signal-to-noise ratio in the data, but our large sample size at all levels (bites, people, and snake species) should still provide sufficient statistical power to provide meaningful results. The full survey is available in Figure S1.

All subjects gave their informed consent for inclusion before they participated in the study. The protocol was approved by the Bangor University, College of Natural Sciences Ethics Committee on 14th November 2016 (CNS2016HWS01).

### 4.2. Data Analysis

Because we were using data across multiple species, we used a phylogenetic comparative approach to investigate the patterns of pain-inducing venoms across species and time [80]. We obtained a phylogeny from the TimeTree database [81] based on the list of species from our dataset. For this purpose (and hence subsequent comparative analyses) five records were removed as they represented unidentified species or hybrids. Thirty-nine species that were present in the dataset but not the TimeTree database were either added manually to the phylogeny at an appropriate position sister to congeneric species (10 species) where possible, or were replaced by ‘phylogenetically equivalent’ species [82] to download the tree (29 species). In the case of phylogenetic equivalent species, we corrected the replacement names to match our dataset after obtaining the phylogeny, so the names in our figures match the dataset. Overall, the final phylogeny and subsequent comparative analyses contained 192 species. Both the phylogeny and list of species, with notes on how species which were present in the dataset, but not the TimeTree database were included, is provided in Table S1. All comparative analyses were conducted in R v3.6.0 [83]. R Code used for analysis is provided in Supplementary Materials.

We used two types of comparative analyses in this study. First, we investigated how our ‘distraction index’ (no pain [‘never’ = pain never became distracting], rapid onset of pain [‘early’ = ≤ 5 min], or delayed onset of pain [‘late’ = > 5 min]) has evolved over the evolutionary history of venomous reptiles using ancestral state estimation. We used Bayesian stochastic mapping [84] implemented in phytools v0.6.99 [85] to estimate ancestral states. Because we had intraspecific variation in our trait of interest (distraction index), we coded our species to include this ‘polymorphism’ with 7 possible states: none, early, late, none+early, none+late, early+late, or none+early+late. This coding also allows us to group these states into those which include early pain onset and those that don’t after the analysis, improving the interpretability of the results in the context of our questions. Ancestral state estimates were based on 1000 simulations under an ‘all rates different’ model for which the transition rates were estimated from the data, as was the prior distribution at the root of the tree.

Second, we investigated how the variation in the magnitude of pain was partitioned for three time periods: immediately (within 1 min of the bite), early (within 5 min of the bite), and throughout the total duration of the bite (i.e., the maximum level of pain experienced). This gives a measure of consistency such that if a large proportion of the variance is attributed to snake species then the pain caused by a particular species should be fairly consistent, but this can vary greatly between snake species. If venom has been selected for a defensive role then we expect that variance explained by individual bites should be low (bites by a given species should be broadly consistent in pain induction). To test this, we constructed three phylogenetic mixed models using MCMCglmm v2.29 [86], one to predict the magnitude of pain (modelled as a Poisson distribution) at each of immediate, early, and maximum time periods. Note that phylogenetic mixed models are not restricted to one datapoint per species, so in addition to allowing us to evaluate intraspecific variation we were also able to use our total dataset of 584 observations for each model. The models included the distraction index as a fixed effect to control for any effects of pain trajectory on the level of pain experienced at a given time point, but inference is primarily based on the random effects included in each model. The random effects were the phylogeny (closely related species cause similar pain levels, distantly related species differ), snake species (pain levels vary based on the particular species that inflicted the bite, regardless of phylogeny), and victim (pain levels vary between people who are bitten). Note that the residual variance in this case can then be considered as an effect of individual bites (pain levels from every individual bite are unique) after accounting for the snake species and clade as well as the person bitten. MCMCglmm uses inverse Wishart prior distributions on parameters and we set all priors to have V = 1 and nu = 0.002. MCMC chains were run for 1.1 million generations, the first 100,000 of which were discarded as burning, and posterior samples were saved every 1000 generations. The quality of each model was checked using autocorrelation plots, Geweke plots, and effective sample sizes (all of which were over 500, mean = 852), and in all cases, models ran well.

We note that the two types of comparative analyses are independent in their interpretations. For instance, even if our mixed models find evidence for much of the variation in pain being due to the individual person bitten, the ancestral state estimations of distraction index are interpretable (despite being concerned mostly with snake species and clade levels). This is because they address two different questions. The mixed models are considering variation in the level of pain experienced, whereas the distraction index (for which we estimate ancestral states) is a measure of the trajectory of the level of pain over the bite. One way to think of this difference is that a bite could start off either mild or very painful and still gradually get worse (or better) over time. Note again that to guard against systematic associations between the level of pain and pain trajectory, our mixed models include the distraction index as a fixed effect (incorporated during the estimation of the variance partitioning of the random effects under examination).

## Figures and Tables

**Table 1:** Distribution of bites received by sex to age of victim at the time of bite.

Age (years)	Male	Female	Unreported	Total
Total, *n* (%)	523 (89.6%)	51 (8.7%)	10 (1.7%)	584 (100%)
11–20, *n* (%)	129 (22.1%)	11 (1.9%)	1 (0.2%)	141 (24.3%)
21–30, *n* (%)	164 (28.1%)	27 (4.6%)	6 (1.0%)	197 (33.7%)
31–40, *n* (%)	102 (17.5%)	7 (1.2%)	1 (0.2%)	110 (18.8%)
41–50, *n* (%)	69 (11.8%)	3 (0.5%)	2 (0.3%)	74 (12.7%)
51–60, *n* (%)	41 (7.0%)	0	0	41 (7.0%)
≥61, *n* (%)	14 (2.4%)	3 (0.5%)	0	17 (2.9%)
Unreported, *n* (%)	4 (0.7%)	0	0	4 (0.7%)

**Table 2:** Estimated transition rates between state combinations of pain trajectories according to the fitted model behind our ancestral state estimates. Rates are given as probabilities of transitioning from the state in the row to the state in the column of the table per million years of evolution. States which include early pain-induction are highlighted in bold. The diagonal is marked with - to signify that there is no transition rate from one state to itself since no change happens in that case.

From\To	None	None+Early	Early	Early+Late	None+Early+Late	None+Late	Late
none	-	0.004	0	0.011	0.003	0	0.041
none+early	0	-	0.032	0	0	0.205	0
early	0	0.125	-	0	0	0	0
early+late	0	0	0	-	0	0.070	0.244
none+early+late	0.057	0	0.016	0	-	0.043	0.008
none+late	0.018	0	0.042	0	0	-	0.111
late	0.141	0	0.032	0.022	0.069	0	-

## Supplementary Materials

The following are available online at https://www.mdpi.com/2072-6651/12/3/201/s1, Figure S1: Pain in Snakebite Questionnaire, R script for Bayesian stochastic mapping and phylogenetic mixed models, Table S1: TimeTree database species list with notes on alterations.
